# Targeting vasculature to reduce fetal growth restriction associated bronchopulmonary dysplasia

**DOI:** 10.1111/resp.14365

**Published:** 2022-09-07

**Authors:** Arvind Sehgal, Beth J. Allison

**Affiliations:** ^1^ Monash Newborn Monash Children's Hospital Clayton Victoria Australia; ^2^ Department of Pediatrics Monash University Clayton Victoria Australia; ^3^ The Ritchie Centre Hudson Institute of Medical Research Clayton Victoria Australia; ^4^ Department of Obstetrics and Gynaecology Monash University Clayton Victoria Australia

**Keywords:** angiotensin converting enzyme, bronchopulmonary dysplasia, reactive oxygen species

**Key points**

1. Premature infants with fetal growth restriction are at a significantly higher risk for developing bronchopulmonary dysplasia.
2. Increased activity of renin–angiotensin–aldosterone system and oxidative stress damage are common mediators.
3. Angiotensin converting enzyme inhibitors and melatonin (antioxidant) are promising therapies, which are physiologically suited to address the disease pathophysiology.

Fetal growth restriction (FGR) affects a significant proportion of pregnancies delivered prematurely. Generally, FGR refers to birthweight <10th centile for gestational age and sex, accompanied by absent/reversed Doppler in systemic arteries. Placental insufficiency (failure to supply adequate oxygen and nutrients to the developing fetus) is the dominant underlying pathology. Premature infants are at increased risk for developing bronchopulmonary dysplasia (BPD), with approximately 20‐fold greater risk of mortality and/or perinatal morbidity in FGR preterm infants. A linear trajectory between higher incidence of BPD and severity of FGR has been demonstrated. FGR increases the risk for BPD; adverse respiratory outcomes attributable to the FGR state being independent of the degree of prematurity. Arrested development of capillaries and alveoli, leading to reduced gas‐exchange surface and dysmorphic pulmonary arteries are hallmarks of ‘new BPD’. Pulmonary hypertension (PH) complicates the management of infants with severe BPD, and is characterized by persistent vasoconstriction and structural remodelling of the pulmonary blood vessels.

Involvement of vasculature has been proposed as an important instigating hypothesis for BPD in FGR infants.^1^ Normal angiogenesis is important for normal alveolarization, and disruption of angiogenesis during critical periods of lung growth (as in utero‐placental insufficiency in FGR) can impair alveolarization, thereby contributing to BPD. Chronic in‐utero hypoxaemia leads to muscularization of precapillary vessels. Overlapping vascular undertones in FGR and BPD cohorts include altered composition of extracellular matrix (in utero disruption in elastin synthesis and its replacement with much stiffer collagen), impaired endothelial function, hyperactivity of the renin–angiotensin–aldosterone system (RAAS), increased reactive oxygen species (ROS) and sympathetic tone and nitric oxide (NO) inhibition. Others include lower vascular endothelial growth factor and thickened blood‐gas barrier, effecting reduced pulmonary compliance and lowering gas‐exchange capacity. High‐resolution ultrasound of the pulmonary arteries has enabled demonstration of thicker and stiffer vasculature in human FGR infants.^1^ Similar impairments in vascular dynamic properties have also been demonstrated in non‐FGR infants who developed severe BPD, attesting to vascular aberrations being an important intersecting link, worthy of being explored for therapeutic potential.^2^

Prominent amongst the mediators are heightened RAAS and ROS, and their status as therapeutic targets is further discussed (RAAS [angiotensin converting enzyme—ACE] and ROS [melatonin]). Figure 1 depicts multifaceted involvement of the RAAS in disease physiology. Angiotensin II is a potent vasoconstrictor of the pulmonary vascular bed. The RAAS plays an important role in hyperoxia‐induced lung injury in neonates. Rodents exposed to hyperoxia in the neonatal period rapidly increased expression of angiotensin II and its receptors well as ACE. Increased RAAS also leads to increased collagen synthesis, which makes the arteries stiffer. Such alterations in the extracellular matrix constitution in pulmonary vasculature contribute to the high pulmonary vascular resistance in BPD. RAAS regulation is crucial to ensure normal lung development, and dysregulation can lead to pathological lung development and increased risk of morbidity such as BPD.^3^ RAAS is upregulated in FGR. In neonatal rats, prophylactic treatment with agonists for MAS oncogene or angiotensin receptor‐2 attenuates cardio‐pulmonary injury by reducing pulmonary inflammation and preventing PH,^4^ indicating benefits in BPD. Effects of ACE inhibitors other than reducing blood pressure (BP) include improved endothelial function, bradykinin mediated vasodilatation, endothelial NO release and change in extracellular matrix, making them physiologically very suited to the proposed FGR‐BPD pathophysiology. They reset the balance between vasoconstrictors/proliferators (ROS, angiotensin II and endothelin‐I) and vasodilators/anti‐proliferators (NO, bradykinin and prostacyclin) in the vessel wall. The balance between the opposing effector molecules angiotensin II (vasoconstrictor) and angiotensin‐(1–7) (vasodilator) may play a pivotal role in lung fibrosis. Improved endothelial function also regulates vascular smooth muscle tone. ACE inhibitors exert significant advantage over other anti‐hypertensive medications. A multicentre study on approximately 300 patients noted that while calcium antagonists, β‐blockers, diuretics and ACE inhibitors, all were equally effective in reducing BP, but only ACE inhibitors improved endothelial function.^5^ In human infants with severe BPD, our group has demonstrated improvement in respiratory support requirements and cardiovascular function, and regression of arterial thickness after a 5‐week administration of captopril.^6^ In summary, this class of drugs may be beneficial in preventing/treating FGR associated BPD and functional lung impairments.

**FIGURE 1:**
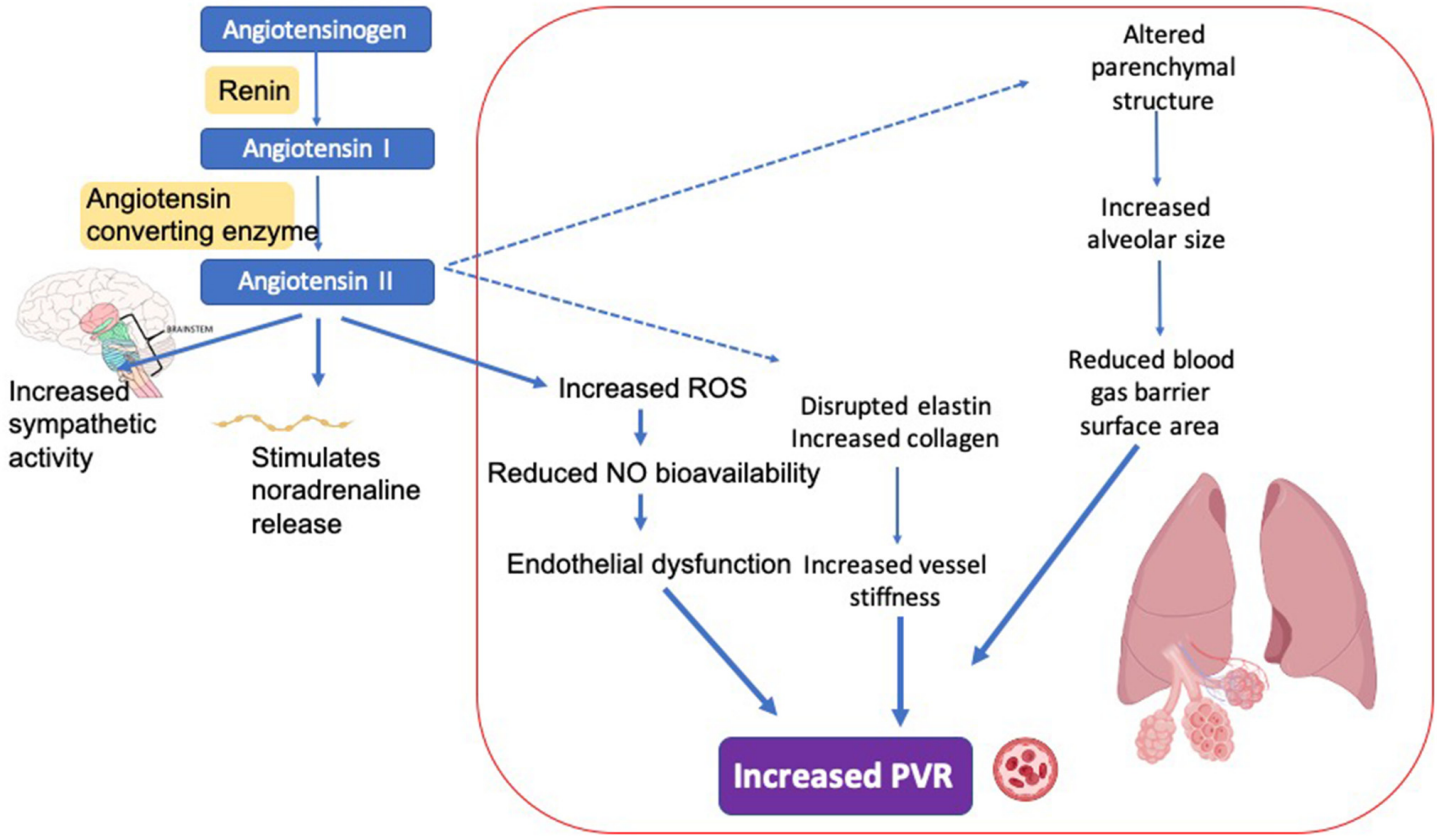
Simplified schematic showing the potential role of the renin–angiotensin–aldosterone system in the development of increased pulmonary vascular resistance (PVR) in bronchopulmonary dysplasia. NO, nitric oxide; ROS, reactive oxygen species. Dashed lines indicate proposed relationships.

Hypoxia/ischemia/inflammation instigated oxidative stress damages the alveolo‐capillary membrane and endothelium. Oxidative stress is involved in the development of BPD in preterm infants (↑ in oxidative damage to lipids and proteins in lung tissue and ↓ levels of antioxidants). Pre‐eclamptic pregnant women have reduced melatonin levels (possibly explaining suppressed antioxidant capacity found in preeclampsia). Melatonin is a broad‐spectrum antioxidant and a potent free radical scavenger, with pulmonary level vasodilator properties. In chronically hypoxic rats, daily administration of melatonin alleviated PH and reversed vascular remodelling, and attenuated right ventricular pressures, and oxidative and inflammatory parameters.^7^ Treatment in chronically hypoxic lambs in the initial days after birth also noted similar results. While physiologically appropriate, data on human cohorts demonstrating effects on pulmonary vasculature are not available.

In conclusion, evidence from experimental models supports the vascular constructs between FGR and BPD. Further refinement in the selection of physiologically appropriate drug and dose, and timing of administration (in utero‐prevention, or postnatal age‐disease severity amelioration), needs prospective analysis.

## CONFLICT OF INTEREST

None declared.
